# Impact on pulmonary, cardiac, and renal function and long-term quality of life after hospitalization for acute respiratory distress syndrome due to COVID-19: Protocol of the Post-COVID Brazil 3 study

**DOI:** 10.62675/2965-2774.20240258-en

**Published:** 2024-06-05

**Authors:** Fernando Luís Scolari, Marciane Maria Rover, Geraldine Trott, Mariana Motta Dias da Silva, Denise de Souza, Raine Fogliati de Carli Schardosim, Rosa da Rosa Minho dos Santos, Emelyn de Souza Roldão, Duane Mocellin, Jennifer Menna Barreto de Souza, Aline Paula Miozzo, Gabriela Soares Rech, Carolina Rothmann Itaqui, Juliana de Mesquita, Gabriel Pozza Muller Estivalete, Hellen Jordan Martins Freitas, Catherine Vitória Pereira dos Santos, Lucas Gobetti da Luz, Marcelo Kern, Milena Soriano Marcolino, Bruna Brandão Barreto, Paulo R. Schwartzman, Ana Carolina Peçanha Antonio, Maicon Falavigna, Caroline Cabral Robinson, Carisi Anne Polanczy, Regis Goulart Rosa

**Affiliations:** 1 Hospital Moinhos de Vento Porto Alegre RS Brazil Project Offices, Hospital Moinhos de Vento - Porto Alegre (RS), Brazil.; 2 Hospital Moinhos de Vento Research Institute Porto Alegre RS Brazil Research Institute, Hospital Moinhos de Vento - Porto Alegre (RS), Brazil.; 3 Hospital Moinhos de Vento Department of Nephrology Porto Alegre RS Brazil Department of Nephrology, Hospital Moinhos de Vento - Porto Alegre (RS), Brazil.; 4 Hospital Moinhos de Vento Department of Internal Medicine Porto Alegre RS Brazil Department of Internal Medicine, Hospital Moinhos de Vento - Porto Alegre (RS), Brazil.; 5 Universidade Federal de Minas Gerais Faculdade de Medicina Department of Clinical Medicine Belo Horizonte MG Brazil Department of Clinical Medicine, Faculdade de Medicina, Universidade Federal de Minas Gerais - Belo Horizonte (MG), Brazil.; 6 Universidade Federal da Bahia Faculdade de Medicina da Bahia Department of Internal Medicine and Diagnostic Support Salvador BA Brazil Department of Internal Medicine and Diagnostic Support, Faculdade de Medicina da Bahia, Universidade Federal da Bahia - Salvador (BA), Brazil.; 7 Hospital Moinhos de Vento Departamento de Cardiologia Porto Alegre RS Brazil Departamento de Cardiologia, Hospital Moinhos de Vento - Porto Alegre (RS), Brazil.; 8 Universidade Federal do Rio Grande do Sul Hospital de Clínicas de Porto Alegre Intensive Care Unit Porto Alegre RS Brazil Intensive Care Unit, Hospital de Clínicas de Porto Alegre, Universidade Federal do Rio Grande do Sul - Porto Alegre (RS), Brazil.; 9 Research Unit, Inova Medical Porto Alegre RS Brasil Research Unit, Inova Medical - Porto Alegre (RS), Brasil

**Keywords:** COVID-19, Coronavirus, SARS-CoV-2, Respiratory distress syndrome, Quality of life, Intensive care units, Brazil

## Abstract

**Rationale:**

Evidence about long-term sequelae after hospitalization for acute respiratory distress syndrome due to COVID-19 is still scarce.

**Purpose:**

To evaluate changes in pulmonary, cardiac, and renal function and in quality of life after hospitalization for acute respiratory distress syndrome secondary to COVID-19.

**Methods:**

This will be a multicenter case–control study of 220 participants. Eligible are patients who are hospitalized for acute respiratory distress syndrome due to COVID-19. In the control group, individuals with no history of hospitalization in the last 12 months or long-term symptoms of COVID-19 will be selected. All individuals will be subjected to pulmonary spirometry with a carbon monoxide diffusion test, chest tomography, cardiac and renal magnetic resonance imaging with gadolinium, ergospirometry, serum and urinary creatinine, total protein, and urinary microalbuminuria, in addition to quality-of-life questionnaires. Patients will be evaluated 12 months after hospital discharge, and controls will be evaluated within 90 days of inclusion in the study. For all the statistical analyses, p < 0.05 is the threshold for significance.

**Results:**

The primary outcome of the study will be the pulmonary diffusing capacity for carbon monoxide measured after 12 months. The other parameters of pulmonary, cardiac, and renal function and quality of life are secondary outcomes.

**Conclusion:**

This study aims to determine the long-term sequelae of pulmonary, cardiac, and renal function and the quality of life of patients hospitalized for acute respiratory distress syndrome due to COVID-19 in the Brazilian population.

## INTRODUCTION

The pulmonary pathology caused by severe acute respiratory syndrome coronavirus 2 (SARS-CoV-2) has resulted in a substantial increase in the demand for ventilatory assistance and long stays in intensive care units (ICUs).^(1)^ The initial data indicate that approximately 15% of patients affected by SARS-CoV-2 require hospitalization, and about 3% will require intensive care.^(2)^ Coronavirus disease 2019 (COVID-19) is estimated to have caused more than 12 million ICU admissions worldwide.^(3)^

Acute respiratory distress syndrome (ARDS) is the most feared pulmonary complication related to COVID-19 due to its association with a long hospital stay and mortality.^(4)^ Approximately 42% of patients with COVID-19 pneumonia develop ARDS, and 60-80% require intensive care.^(5)^ Acute respiratory distress syndrome is an inflammatory process resulting from local or systemic injury to the pulmonary alveolar-capillary membrane, causing an increase in local vascular permeability, with subsequent interstitial and alveolar edema.^(6)^ Even if patients are discharged from the hospital, ARDS survivors are at high risk of persistent physical disability.^(7)^ Prepandemic studies have shown that ARDS survivors have reduced muscle function, with a high prevalence of physical-functional disability, restrictive ventilatory disorder, and decreased pulmonary diffusion.^(8–10)^ In one study 39% of ARDS survivors exhibited critically ill polyneuropathy, their physical weakness lasting for up to 12 months after hospital discharge.^(7)^ Despite the predominant respiratory involvement, several reports have identified additional direct or indirect involvement of the cardiovascular and renal systems.^(11–14)^ These acute insults can lead to long-term organ dysfunction, reducing quality of life and necessitating follow-up and treatment of sequelae over a longer period.^(15)^

According to the World Health Organization,^(16)^ detecting any causal link between COVID-19 and long-term sequelae is a research priority. Evidence on the prevalence of pulmonary, cardiovascular, and renal sequelae in survivors of ARDS due to COVID-19 in the Brazilian population is still scarce. We present the study protocol for the Post-COVID-19 Brazil 3 study, with a focus on analyzing the impact on pulmonary, cardiac, and renal functions, as well as quality of life, among ARDS survivors with COVID-19 starting 12 months after discharge.

## METHODS

**Figure 1:**
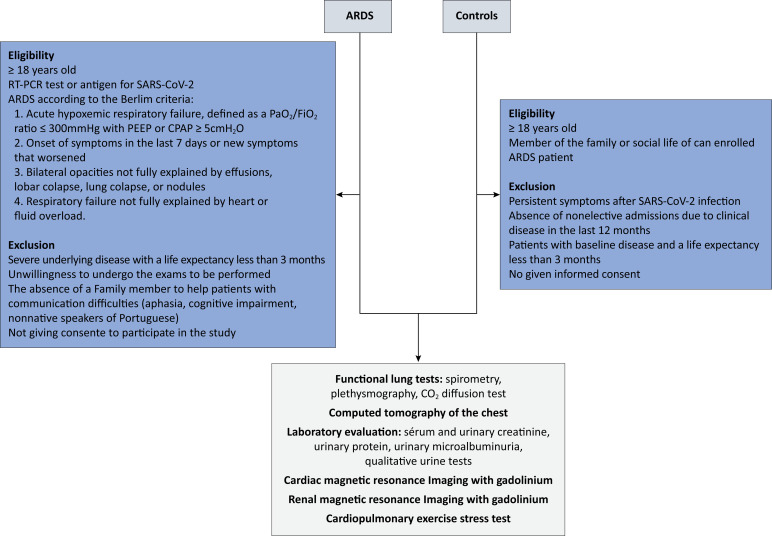
Study design.

This is a prospective, multicenter case–control study of patients who survived hospitalization for COVID-19 and were diagnosed with ARDS and controls without persistent symptoms of COVID-19 and hospitalization due to clinical disease in the last 12 months. The study design is summarized in figure 1. The study protocol is registered at Clinicaltrials.gov (NCT05225194). This study was approved by the National Research Ethics Committee (Conep) (CAAE: 54943222.7.1001.5330) and adheres to resolution 466/12 of the National Health Council (CNS) of Brazil. Free and informed consent will be obtained from all participants.

ARDS - acute respiratory distress syndrome; RT-PCR - polymerase chain reaction after reverse transcription; SARS-CoV-2 - severe acute respiratory syndrome coronavirus 2; PaO_2_ - partial pressure of oxygen; FiO_2_ - fraction of inspired oxygen; PEEP - positive end-expiratory pressure; CPAP - continuous positive airway pressure; CO_2_ - carbon monoxide.

### Patient selection

#### Patients with acute respiratory distress syndrome

Patients will be considered eligible if they are ≥ 18 years of age, have a positive reverse transcription polymerase chain reaction (RT-PCR) test or a positive antigen test for SARS-CoV-2 infection collected via nasopharyngeal swab during hospitalization or within 14 days beforehand, and are diagnosed with ARDS due to COVID-19 during hospitalization, and hospital discharge from the index hospitalization. The diagnosis of ARDS was defined according to the Berlin criteria:^(17)^ respiratory failure not fully explained by heart failure or fluid overload; onset within one week of the known lesion or new symptoms that worsened; bilateral opacities not fully explained by effusions, lobar collapse, lung collapse, or nodules; hypoxemia defined as the relationship between partial pressure of oxygen and fraction of inspired oxygen (PaO_2_/FiO_2_) ≤ 300mmHg, with positive end-expiratory pressure or continuous positive airway pressure ≥ 5cmH_2_O. The exclusion criteria are severe underlying disease with a life expectancy less than 3 months, unwillingness to undergo the tests to be performed, absence of a family member if the patient has communication difficulties (aphasia, cognitive impairment, nonnative speakers of Portuguese), and nonconsent to participate in the study.

#### Controls

Individuals ≥ 18 years of age who are family members or social interactors of participants who have survived ARDS due to COVID-19 will be considered controls. Individuals who are unwilling to undergo the proposed tests, have a history of persistent symptoms after SARS-CoV-2 infection, have a history of nonelective hospitalization due to clinical disease in the last 12 months, have a severe underlying disease with a life expectancy of less than 3 months, or do not consent to participate in the study will be ineligible. The following symptoms are considered persistent symptoms of COVID-19: cough, fatigue, muscle pain, dyspnea, anosmia, ageusia, and memory or concentration deficit, as previously evaluated by a physician and attributed to COVID-19.

### Recruitment

The patients will be recruited from hospitals located in the city of Porto Alegre and its metropolitan region. Patients with ARDS will be accessed from outpatient clinics, databases, or previous records of ICU admission due to ventilatory failure of patients undergoing clinical follow-up at the respective centers, and controls will be recruited from family members or people living with the patients. Telephone contacts will be made after hospital discharge, and the eligibility criteria and the free and informed consent form will be applied. With duly informed and signed consent, the following procedures will be performed: a review of medical records and structured telephone interviews and teleconsultation to assess eligibility and scheduling of previously specified exams.

### Procedures

**Figure 2:**
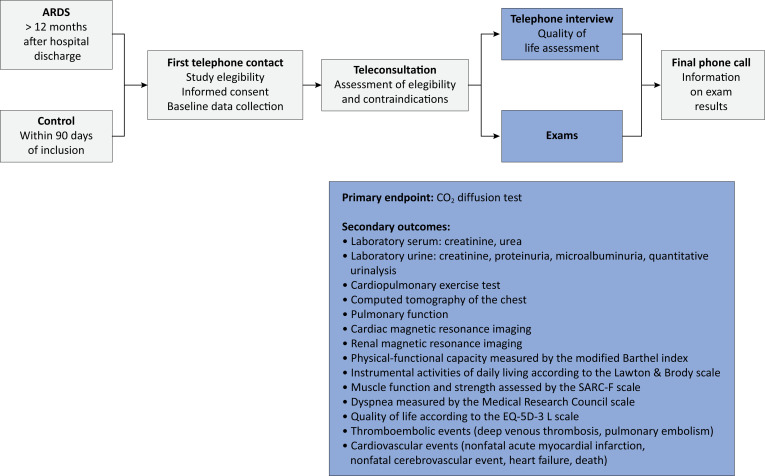
Trajectory of the study participants.

All participants will undergo teleconsultation by telephone to evaluate possible contraindications for the following complementary exams: serum creatinine and urea, creatinine and total protein in urine, microalbuminuria and qualitative urinalysis; spirometry with pulmonary diffusion for carbon monoxide (CO_2_) without a bronchodilator test; treadmill ergospirometry with a ramp protocol; high-resolution tomography of the chest (noncontrast); and cardiac and renal magnetic resonance imaging with gadolinium contrast. Patients with contraindications to one or more exams who are eligible will remain in the study. Teleconsultation and exams will be performed between 12 and 36 months after discharge from the index hospitalization for ARDS or, for controls, at the time of inclusion (with a restricted window of up to 90 days due to the risk of controls developing COVID-19 or other clinical complications after the initial visit). Figure 2 is the flow chart of the participants in the study.

ARDS - acute respiratory distress syndrome; CO_2_ - carbon monoxide; EQ-5D-3 L - EuroQol 5 Dimensions 3 Levels.

### Outcomes

The primary outcome of the study is the pulmonary diffusing capacity for carbon monoxide (DLCO) measured after 12 months. The following were considered secondary outcomes: physical-functional capacity measured by the modified Barthel index after 12 months;^(18)^ instrumental activities of daily living measured by the Lawton and Brody scale of instrumental activities of daily living from 12 months onward;^(19)^ muscle function and strength assessed by Strength, Assistance with Walking, Rising from a Chair, Climbing Stairs, and Falls (SARC-F) at 12 months of age;^(20)^ dyspnea measured by the modified *Medical Research Council* (MRC) dyspnea scale from 12 months of age;^(21)^ health-related quality of life assessed using the EuroQol 5 Dimensions 3 Levels questionnaire (EQ-5D-3L) from 12 months onward;^(22.23)^ ergospirometric variables from 12 months onward: direct oxygen consumption, maximum oxygen consumption (VO_2_ peak), minute volume/CO_2_ production ratio (VE/VCO_2_ slope) and oxygen uptake efficiency (OUES - Oxygen Uptake Efficiency Slope); spirometric variables from 12 months onward: vital capacity (VC), forced expiratory volume in one second (FEV1) and FEV1/VC; and radiological patterns of pulmonary fibrosis assessed by high-resolution tomography of the chest from 12 months onward; radiological patterns related to the sequelae of acute myocarditis assessed by cardiac magnetic resonance imaging with T1- and T2-weighted parametric maps and by means of the delayed enhancement technique from 12 months onward; radiological patterns of sarcopenia assessed by high-resolution tomography of the chest from 12 months onward; changes in renal function or structure assessed by magnetic resonance imaging and laboratory tests; and major cardiovascular events (non-fatal infarction, non-fatal stroke, acute heart failure) and thromboembolic events (deep venous thrombosis, pulmonary embolism) after hospital discharge. The data collection protocol for the exams collected is described in the Supplementary Material.

### Selection of centers

The participating centers will be selected after their representatives complete a questionnaire to assess feasibility. Institutions that demonstrate high potential for recruiting research participants and are in line with good clinical practice will be included, according to the following characteristics: being a reference hospital for the care of patients with COVID-19 in the metropolitan region of Porto Alegre; having an intensive care unit (10 or more beds) for the treatment of COVID-19 patients; demonstrating feasibility for the diagnosis of COVID-19 by RT-PCR or antigen testing; and accepting participation in the study by signing a cooperation agreement.

### Recruitment

The participating centers will select patients whose internal records show that they meet the inclusion criteria for participation in the study. Recruitment will be performed by face-to-face or remote interviews with the patient and/or his or her family by the research team. After they sign the informed consent form and after collection of their baseline data, the participants will be evaluated through a structured and centralized telephone interview. For ARDS/COVID-19 patients, the interview will be conducted 12 months after hospital discharge, and controls will be evaluated within 90 days after their inclusion in the study.

The participants will be included by applying the eligibility criteria, which will be recorded in the triage log. Patients who meet all the inclusion criteria and no exclusion criteria will be invited to voluntarily participate in the study by signing the informed consent form in person or electronically. The process of obtaining informed consent will be detailed in the ethics and good clinical practices section. The participant will be approached by a researcher duly trained in good clinical practice, who will maintain the anonymity and confidentiality of the data and will promote conditions of comfort for the participant.

### Data collection

At baseline, structured interviews will be applied to the study participants, and we will review their medical records to collect sociodemographic data, previous health information, and lifestyle habits before their inclusion in the study. For participants with ARDS, we will collect data on hospitalization for COVID-19, including variables related to severity (sequential organ failure assessment [SOFA], use of mechanical ventilation) and prescribed treatments (vasopressors, corticosteroids).

### Follow-up

All participants will be monitored and evaluated through a structured telephone interview, teleconsultation, and clinical examinations in those without contraindications. This monitoring will be performed by telephone contact with researchers from the coordinating center, which is *Hospital Moinhos de Vento*. The researchers received training for data collection in this modality. The procedures of this stage will be performed in a single telephone exchange located at *Hospital Moinhos de Vento*. Electronic tools (e-mail and WhatsApp) may be used exclusively for scheduling the interviews. Only data on vital status and refusal to follow up will be collected digitally. Medical teleconsultation with the participants will evaluate possible contraindications to undergoing the study exams. After performing the complementary exams, all participants will come to a final teleconsultation that will inform the patients of their exam results and the relevant guidelines for each patient.

Data will be collected using electronic medical records accessible via smartphones, tablets, or computers. The process of digitally collecting and managing data has several benefits over the nonautomated process, such as standardization, reliability, and security of the data collected. The tool used for data collection and management will be the Research Electronic Data Capture (REDCap; https://www.redcapbrasil.com.br/).^(24)^ This data platform will be accessed with a personal and nontransferable username and password, by a member of the team, after due delegation in the study by the principal investigator. Users of the platform (research team) will have specific permissions related to their role and delegation in the study.

### Data security

Several procedures will be employed to ensure the security and quality of the data. All researchers will participate in a training session on good clinical practices and study procedures, including data collection. The researchers will be able to contact the coordinating center of the study to resolve questions or problems that may arise. All processes related to data management complied with the General Data Protection Act (law 13,709 of August 14, 2018). All servers, domain name systems, and means of providing access will be built and maintained in Brazil. This infrastructure will be dedicated to the present study. Therefore, all data will be isolated from any other access, ensuring greater security. In addition, the fact that the servers are headquartered in Brazil will subject the custody of these data to Brazilian law. Digital certificates will also be used for encryption to ensure the security of communications.

The database backup process will be performed automatically every 12 hours. The extraction of data for the statistical software will be done in an automated manner, with data anonymization for checking data consistency, remote monitoring actions, the development of derived variables, and statistical analysis. Data cleaning to identify inconsistencies will be conducted periodically. The researchers will be notified of inconsistencies so that they can correct them. The telephone interviews will be recorded and audited to verify the consistency of the data. The audio files will be stored anonymously on a server with the same security system as the database described. Access to these files will be protected by the research team using a personal, nontransferable username and password. The coordinating center will review monthly detailed reports on triage, inclusion, follow-up, and consistency and completeness of the data. The researchers will immediately take action to resolve any issues. Statistical techniques for fraud identification will be performed throughout the study.

### Calculation of sample size

A sample size of 198 individuals (99 for each group) was calculated to detect a minimum clinically relevant difference of 2mL/minute/mmHg in the mean DLCO between the COVID-19 ARDS patients and healthy controls. Factoring in a 10% rate of losses and refusals, we will need 110 in each group given a desired power of 80%, a significance level of 5%, and a standard deviation of 5mL/minute/mmHg.

### Statistical analysis

The variables will be described as n (%) for qualitative variables, mean ± standard deviation for symmetric quantitative variables, and median (interquartile range) for asymmetric quantitative variables. The distribution of quantitative variables will be assessed by graphical visualization of histograms and the Shapiro–Wilk test. The primary outcome will be compared between the study groups using logistic regression adjusted for sex, age, and comorbidities. The reported measure of effect will be the odds ratio (OR) with its 95% confidence interval (95%CI). The secondary outcomes with qualitative distributions will be evaluated in the same way as the primary outcome. The quantitative distribution of secondary outcomes will be evaluated by means of multiple linear regression adjusted for sex, age, and comorbidities. The reported measure of effect will be the difference in means with their 95%CIs. P values < 0.05 will be considered statistically significant. Secondary outcomes will not be adjusted for multiple variables. Thus, the findings for the secondary outcomes will be interpreted as exploratory. The analyses will be performed with R software packages to be detailed at the time of analysis.^(25)^

### Ethics and good clinical practices

The study was planned and will be conducted in accordance with CNS Resolution 466 of December 12, 2012^(26)^ and the Guidelines for Good Clinical Practice, Amendment 6 - Revision 2 of the International Council for Harmonization,^(27)^ in addition to the standards recommended by the General Data Protection Act. The study will begin only after full approval of the protocol and related documents by the Research Ethics Committee/Conep system at the study sites.

### Consent to participate

Following the Regulatory Guidelines and Norms for Research Involving Humans, established by CNS Resolution 466/12, informed consent will be obtained from the research participants at the time of invitation to participate in the study. The informed consent form will be prepared in a way that provides, in accessible and clear language, information about the study's objectives, methods, process of collection, and registration. The informed consent form may be applied face to face (to patients undergoing a review appointment) or electronically.

### Dissemination

The researchers will submit the results of the study at scientific conferences and meetings and will write a manuscript that will be submitted for publication in a peer-reviewed journal. The study steering committee will participate in the analysis of the results, in the preparation of the manuscript, and in the selection of the journal for publication. Authorship criteria will be defined according to the International Committee of Medical Journal Editors (ICMJE).

### Data sharing

The authors encourage researchers to contact the corresponding author to access and share unpublished data. The steering committee will have a role in judging and providing the requested data.

## DISCUSSION

Little is known about the long-term sequelae among survivors of ARDS caused by COVID-19, and the magnitude of these sequelae may impact the planning and allocation of resources by health systems. Data from an Italian cohort of patients admitted to the ICU for ARDS in the first half of 2020 showed that among the survivors, one-third showed signs of fatigue and loss of muscle strength 3 months after discharge, followed by improvement in the physical component of quality of life after 12 months.^(7)^ The pulmonary evaluation performed 3 months after discharge in another cohort revealed a high prevalence of functional and structural lung changes.^(28)^ However, these data mostly come from European or North American cohorts with severe COVID-19 infection, especially of patients included in the first waves of the disease, before vaccination of the population. The prevalence of physical and mental health sequelae among Brazilian ARDS survivors due to COVID-19 is still unknown.

Cardiovascular events have been reported during the acute phase of COVID-19,^(13)^ but the risk may persist after the infection resolves. A population-based study suggested that individuals are at risk of cardiovascular events such as stroke, arrhythmias, acute coronary syndrome, myocarditis, and pericarditis up to 1 year after the acute phase.^(14)^ Cardiac magnetic resonance imaging has demonstrated a high incidence of post-COVID-19 myocardial changes, but most of these studies included outpatients with a follow-up of only 4 months.^(29)^ Our study aimed to determine the prevalence of long-term cardiac sequelae, such as ventricular dilation and loss of cardiac function, among individuals with severe COVID-19. Furthermore, myocardial fibrosis detected by the delayed gadolinium enhancement technique may be related to prior myocarditis or ischemic events in the acute phase of COVID-19. Its detection is correlated with arrhythmic events and a worse prognosis,^(30)^ and it is important in the stratification of the severity of these individuals.

Acute kidney injury is a common complication in the acute phase of COVID-19 and is associated with worse outcomes and progression to chronic kidney disease, especially in those with greater severity at presentation.^(12)^ The factors that may be associated with kidney injury caused by COVID-19 include viral agent-mediated endothelial damage, complement activation, local inflammation, and glomerulopathy,^(11)^ as well as other factors such as hemodynamic status in acute infection, nephrotoxic medication, and thrombotic events, among other indirect agents.^(31)^ Long-term kidney damage may be related not only to the direct maintenance of the offending agent but also indirectly to the exacerbation of cardiovascular diseases that lead to greater kidney damage.^(11)^ We hope to estimate the long-term prevalence of renal damage associated with COVID-19 in the Brazilian population by measuring kidney function and examining its structure.

This study will have several strengths. It is a multicenter study that will prospectively evaluate long-term sequelae. Many of the previous studies that investigated long-term COVID-19 and its sequelae included patients shortly after acute infection.^(13.28)^ Due to the few cases and the severity of COVID-19, the patients included may have come from the earlier stages of the pandemic. In addition, the retrospective collection of data regarding index hospitalization may limit our study.

## Supplementary Material




