# Supplementary material for: Impact on pulmonary, cardiac, and renal function and long-term quality of life after hospitalization for acute respiratory distress syndrome due to COVID-19: Protocol of the Post-COVID Brazil 3 study
Source: Crit Care Sci. 2024 Jun 5;36:e20240258en. doi: 10.62675/2965-2774.20240258-en (PMC11152444; doi:10.62675/2965-2774.20240258-en)
Supplement: Supplementary file 1 [file 2965-2774-ccsci-36-e20240258en-supp01.pdf]

Critical Care Science
https://doi.org/10.62675/2965-2774.20240258-en

SUPPLEMENTARY MATERIAL

Impact on pulmonary, cardiac, and renal function and long-term quality of life after hospitalization for acute respiratory distress syndrome due to COVID-19: Protocol of the Post-COVID Brazil 3 study

Fernando Luís Scolari[1], Marciane Maria Rover[1], Geraldine Trott[1], Mariana Motta Dias da Silva[2], Denise de Souza[1], Raine Fogliati de Carli Schardosim[1], Rosa da Rosa Minho dos Santos[1], Emelyn de Souza Roldão[1], Duane Mocellin[1], Jennifer Menna Barreto de Souza[1], Aline Paula Miozzo[1], Gabriela Soares Rech[1], Carolina Rothmann Itaqui[1], Juliana de Mesquita Neto[1], Gabriel Pozza Muller Estivalete[1], Hellen Jordan Martins Freitas[1], Catherine Vitória Pereira dos Santos[1], Lucas Gobetti da Luz[3], Marcelo Kern[4], Milena Soriano Marcolino[5], Bruna Brandão Barreto[6], Paulo R. Schwartzman[7], Ana Carolina Peçanha Antonio[8], Maicon Falavigna[9], Caroline Cabral Robinson[1], Carisi Anne Polanczy[7], Regis Goulart Rosa[1]

Data collection form for the complementary exams

**Exam results**	
ID	
**Participant information**	
Name	[baseline data_arm_1][part name]
Sex	[baseline data_arm_1][sex]
**Patient released for the following exams**	
Spirometry	[teleconsultation_1_arm_1][spiro]
Ergospirometry	[teleconsultation_1_arm_1][ergo]
Resonance	[teleconsultation_1_arm_1][reso]
Tomography	[teleconsultation_1_arm_1][tomo]
Term of Consent in person	
**Spirometry**	
Was spirometry performed?	☐ Yes<br>☐ No
Reason for nonperformance	☐ Prespecified contraindication<br>☐ Another reason
What other reason?	....
Date of exam	....
VEF1 (L)	....
VEF1 (% do predicted)	....
VEF1/CVF (% do predicted)	....
Forced vital capacity	....
Forced vital capacity (%)	....
Total lung capacity (% of predicted)	....
Residual volume (L)	....
Residual volume (% of predicted)	....
Diffusion of carbon monoxide (DLCO)	....
DLCO/VA (%)	....

Continue...

This is an open access article under the CC BY license (https://creativecommons.org/licenses/by/4.0/).

...continuation

**Ergospirometry**	
Was ergospirometry performed?	☐ Yes<br>☐ No
Reason for nonperformance	☐ Prespecified contraindication<br>☐ Another reason
What other reason?	....
Exam date	....
Peak VO_2 (mL/kg/minute)	....
Peak VO_2 (L/minute)	....
VE/VCO_2 slope	....
RER or Rq or equivalent resp (Rpeak)	....
O_2 saturation drop	☐ Yes<br>☐ No
Lowest value (%)	....
Peak oxygen pulse (PuO_2) VO_2/HR (mL/beat)	....
Resting HR (bpm)	....
Peak HR (bpm)	....
Peak HR (% of predicted)	....
Resting SAP (mmHg)	....
Resting DBP (mmHg)	....
SBP peak exertion (mmHg)	....
DBP peak exertion (mmHg)	....
Identified thresholds	☐ Yes<br>☐ No
VO_2 at the first ventilatory threshold	....
HR at the first ventilatory threshold	....
VO_2 at the second threshold	....
HR at the second threshold	....
Presence of periodic ventilation	☐ Yes<br>☐ No
OUES	....
$PetCO_2$ at rest (mmHg)	....
Was the exam interrupted?	☐ Yes<br>☐ No
Reason for interruption of the exam	☐ General fatigue<br>☐ Fatigue in lower limbs<br>☐ Angina<br>☐ Dyspnea<br>☐ Dizziness<br>☐ Others
**Dynamic magnetic resonance imaging (nonstress)**	
Dynamic (nonstress) magnetic resonance imaging performed?	☐ Yes<br>☐ No
Reason for nonperformance	☐ Prespecified contraindication<br>☐ Another reason
What other reason?	....
Exam date	....
Left ventricular (LV) ejection fraction (%)	....

Continue...

...continuation

LV end-systolic volume	....
LV end-diastolic volume (indexed to BS) (mL/m^2)	....
Effective LV volume (mL/m$_2$)	....
Left ventricular mass (index) (g/m^2)	....
Right ventricular (RV) ejection fraction (%)	....
RV end-diastolic volume	....
RV end-systolic volume	....
RV stroke volume (mL/m^2)	....
RV volume (mL/m^2)	....
Map of T1 (ms)	....
Map of T2 (ms)	....
Pericardial delayed enhancement	☐ Yes<br>☐ No
Pericardial effusion (> 10 mm)	☐ Yes<br>☐ No
Delayed enhancement (fibrosis)	☐ Yes<br>☐ No
Standard	☐ Ischemic<br>☐ Nonischemic
% of delayed enhancement (fibrosis)	....
**Abdominal magnetic resonance imaging**	
Was abdominal magnetic resonance imaging performed?	☐ Yes<br>☐ No
Kidney size (cm)	.... cm
Right kidney size	.... cm
Left kidney size	.... cm
Parenchyma thickness measured in the axial plane (avoiding scarring) (cm)	.... cm
Right kidney	.... cm
Left kidney	.... cm
Kidney volume (based on measurements and calculation of the volume as an ellipse – multiplier constant 0.52)	.... cm
Right kidney	……….......................... cm^3
Left kidney	……….......................... cm^3
Pattern of contrast uptake in the venous phase	☐ Homogeneous<br>☐ Heterogeneous<br>☐ Focal change suggestive of scarring
Signal strength standard	☐ Homogeneous<br>☐ Heterogeneous<br>☐ Focal change (do not include cysts or solid lesions in this item)
Morphology of pyelocaliceal systems	☐ Normal<br>☐ Diffuse and bilateral hydronephrosis<br>☐ Diffuse, unilateral hydronephrosis<br>☐ Focal caliectasis
Comparative analysis with examination before admission?	☐ Yes<br>☐ No
Considerations	☐ Stable renal dimensions<br>☐ Reduction in the size of the kidneys<br>☐ Emergence of focal change/lesion

Continue...

...continuation

**High-resolution chest tomography (noncontrast)**	
Was high-resolution tomography of the chest (noncontrast) performed?	▢ Yes<br>▢ No
Reason for nonperformance	▢ Prespecified contraindication<br>▢ Another reason
What other reason?	....
Exam date	....
Is there involvement of the lung parenchyma?	▢ Yes<br>▢ No
Percentage of involvement	▢ Less than 25%<br>▢ 25% a 50%<br>▢ 51% a 75%<br>▢ > 75%
Type of lung parenchyma involvement	▢ Infiltrated in frosted glass<br>▢ Fibrosing interstitial pneumonia<br>▢ Fibrous scar<br>▢ Consolidation<br>▢ Emphysema<br>▢ Other ....
Emphysema level	▢ Lightweight<br>▢ Moderate<br>▢ Severe
**Evaluation of sarcopenia**	
What is the unit of measurement for the cross-sectional area?	▢ cm^2<br>▢ mm^2
What is the cross-sectional area of the pectoral muscles (measure immediately below the aortic arch)	....
Mean muscle density at this level (Hounsfield units)	....
Coronary calcifications?	▢ Yes<br>▢ No<br>▢ Not evaluated
