# Supplementary material for: Impact on pulmonary, cardiac, and renal function and long-term quality of life after hospitalization for acute respiratory distress syndrome due to COVID-19: Protocol of the Post-COVID Brazil 3 study
Source: Crit Care Sci. 2024 Jun 5;36:e20240258en. doi: 10.62675/2965-2774.20240258-en (PMC11152444; doi:10.62675/2965-2774.20240258-en)
Supplement: Supplementary file 2 [file 2965-2774-ccsci-36-e20240258en-pt-supp01.pdf]

MATERIAL SUPLEMENTAR

Impacto nas funções pulmonar, cardíaca e renal e na qualidade de vida a longo prazo após internação por síndrome do desconforto respiratório agudo por COVID-19: protocolo do Estudo 3 Pós-COVID Brasil

Fernando Luís Scolari[1], Marciane Maria Rover[1], Geraldine Trott[1], Mariana Motta Dias da Silva[2], Denise de Souza[1], Raine Fogliati de Carli Schardosim[1], Rosa da Rosa Minho dos Santos[1], Emelyn de Souza Roldão[1], Duane Mocellin[1], Jennifer Menna Barreto de Souza[1], Aline Paula Miozzo[1], Gabriela Soares Rech[1], Carolina Rothmann Itaqui[1], Juliana de Mesquita Neto[1], Gabriel Pozza Muller Estivalete[1], Hellen Jordan Martins Freitas[1], Catherine Vitória Pereira dos Santos[1], Lucas Gobetti da Luz[3], Marcelo Kern[4], Milena Soriano Marcolino[5], Bruna Brandão Barreto[6], Paulo R. Schwartzman[7], Ana Carolina Peçanha Antonio[8], Maicon Falavigna[9], Caroline Cabral Robinson[1], Carisi Anne Polanczy[7], Regis Goulart Rosa[1]

Ficha de coleta de dados dos exames complementares

**Resultados dos exames**	
ID	
**Informações do participante**	
Nome	[dados_basais_arm_1][nome_partic]
Sexo	[dados_basais_arm_1][sexo]
**Paciente liberado para os seguintes exames**	
Espirometria	[teleconsulta_1_arm_1][espiro]
Ergoespirometria	[teleconsulta_1_arm_1][ergo]
Ressonância	[teleconsulta_1_arm_1][resso]
Tomografia	[teleconsulta_1_arm_1][tomo]
Termo de Consentimento presencial	
**Espirometria**	
Espirometria foi realizada?	☐ Sim<br>☐ Não
Motivo da não realização	☐ Contraindicação pré-especificada<br>☐ Outro motivo
Qual outro motivo?	....
Data do exame	....
VEF1 (L)	....
VEF1 (% do previsto)	....
VEF1/CVF (% do previsto)	....
Capacidade vital forçada	....
Capacidade vital forçada (%)	....
Capacidade pulmonar total (% do previsto)	....
Volume residual (L)	....
Volume residual (% do previsto)	....
Difusão de monóxido de carbono (DLCO)	....
DLCO/VA (%)	....

Continua...

Este é um artigo de acesso aberto sob licença CC BY (https://creativecommons.org/licenses/by/4.0/).

...continuação

**Ergoespirometria**	
Ergoespirometria foi realizada?	☐ Sim<br>☐ Não
Motivo da não realização	☐ Contraindicação pré-especificada<br>☐ Outro motivo
Qual outro motivo?	....
Data do exame	....
VO_2 pico (mL.kg.minuto)	....
VO_2 pico (L/minuto)	....
VE/VCO_2 slope	....
RER ou Rq ou equivalente resp (Rpico)	....
Saturação de O_2 queda	☐ Sim<br>☐ Não
Menor valor (%)	....
Pulso de oxigênio de pico (PuO_2) VO_2/FC (mL/batimento)	....
FC repouso (bpm)	....
FC pico (bpm)	....
FC pico (% do previsto)	....
PAS repouso (mmHg)	....
PAD repouso (mmHg)	....
PAS pico do esforço (mmHg)	....
PAD pico do esforço (mmHg)	....
Limiares identificados	☐ Sim<br>☐ Não
VO_2 no primeiro limiar ventilatório	....
FC no primeiro limiar ventilatório	....
VO_2 no segundo limiar	....
FC no segundo limiar	....
Presença de ventilação periódica	☐ Sim<br>☐ Não
OUES	....
$PetCO_2$ em repouso (mmHg)	....
O exame foi interrompido?	☐ Sim<br>☐ Não
Motivo da interrupção do exame	☐ Fadiga geral<br>☐ Fadiga em membros inferiores<br>☐ Angina<br>☐ Dispneia<br>☐ Tontura<br>☐ Outros
**Ressonância magnética estudo dinâmico (sem estresse)**	
Ressonância magnética estudo dinâmico (sem estresse) foi realizada?	☐ Sim<br>☐ Não
Motivo da não realização	☐ Contraindicação pré-especificada<br>☐ Outro motivo
Qual outro motivo?	....
Data do exame	....
Fração de ejeção do ventrículo esquerdo (VE) (%)	....

Continua...

...continuação

Volume sistólico final de VE	....
Volume distólico final de VE (indexado para SC) (mL/m^2)	....
Volume efetivo de VE (mL/m$_2$)	....
Massa ventricular esquerda (index) (g/m^2)	....
Fração de ejeção do ventrículo direito (VD) (%)	....
Volume diastólico final de VD	....
Volume sistólico final de VD	....
Volume ejetivo de VD (mL/m^2)	....
Volume do VD (mL/m^2)	....
Mapa de T1 (ms)	....
Mapa de T2 (ms)	....
Realce tardio pericárdico	☐ Sim<br>☐ Não
Derrame pericárdico (> 10mm)	☐ Sim<br>☐ Não
Realce tardio (fibrose)	☐ Sim<br>☐ Não
Padrão	☐ Isquêmico<br>☐ Não isquêmico
% de realce tardio (fibrose)	....
**Ressonância magnética de abdome**	
Ressonância magnética de abdome foi realizada?	☐ Sim<br>☐ Não
Tamanho dos rins (cm)	.... cm
Tamanho do rim direito	.... cm
Tamanho do rim esquerdo	.... cm
Espessura do parênquima medida no plano axial (evitando cicatrizes) (cm)	.... cm
Rim direito	.... cm
Rim esquerdo	.... cm
Volume dos rins (com base nas medidas e cálculo do volume como elipse – constante multiplicadora 0,52)	.... cm
Rim direito	.... cm^3
Rim esquerdo	.... cm^3
Padrão de impregnação pelo meio de contraste na fase venosa	☐ Homogêneo<br>☐ Heterogêneo<br>☐ Alteração focal, sugestiva de cicatriz
Padrão da intensidade de sinal	☐ Homogêneo<br>☐ Heterogêneo<br>☐ Alteração focal (não incluir cistos ou lesões sólidas nesse item)
Morfologia dos sistemas pielocaliciais	☐ Normal<br>☐ Hidronefrose difusa e bilateral<br>☐ Hidronefrose difusa e unilateral<br>☐ Caliectasia focal
Análise comparativa com exame prévio da internação?	☐ Sim<br>☐ Não
Considerações	☐ Dimensões renais estáveis<br>☐ Redução das dimensões dos rins<br>☐ Surgimento de alteração/lesão focal

Continua...

...continuação

**Tomografia de tórax de alta resolução (SEM contraste)**	
Tomografia de tórax de alta resolução (SEM contraste) foi realizada?	▢ Sim<br>▢ Não
Motivo da não realização	▢ Contraindicação pré-especificada<br>▢ Outro motivo
Qual outro motivo?	....
Data do exame	....
Há presença de acometimento do parênquima pulmonar?	▢ Sim<br>▢ Não
Percentual de acometimento	▢ Inferior a 25%<br>▢ 25% a 50%<br>▢ 51% a 75%<br>▢ > 75%
Tipo de acometimento do parênquima pulmonar	▢ Infiltrado em vidro fosco<br>▢ Pneumonia intersticial fibrosante<br>▢ Cicatriz fibrosante<br>▢ Consolidação<br>▢ Enfisema<br>▢ Outro ....
Nível do enfisema	▢ Leve<br>▢ Moderado<br>▢ Grave
**Avaliação de sarcopenia**	
Qual unidade de medida da área de secção transversal?	▢ cm^2<br>▢ mm^2
Qual a área de secção transversal da musculatura peitoral (mensurar imediatamente abaixo do arco aórtico)	....
Densidade muscular média nesse nível (unidades Housefield)	....
Calcificações coronarianas?	▢ Sim<br>▢ Não<br>▢ Não avaliado
