# Supplementary material for: Impact on pulmonary, cardiac, and renal function and long-term quality of life after hospitalization for acute respiratory distress syndrome due to COVID-19: Protocol of the Post-COVID Brazil 3 study
Source: Crit Care Sci. 2024 Jun 5;36:e20240258en. doi: 10.62675/2965-2774.20240258-en (PMC11152444; doi:10.62675/2965-2774.20240258-en)
Supplement: Translation

# Translation: Impacto nas funções pulmonar, cardíaca e renal e na qualidade de vida a longo prazo após internação por síndrome do desconforto respiratório agudo por COVID-19: protocolo do Estudo 3 Pós-COVID Brasil

**DOI:** 10.62675/2965-2774.20240258-pt

**Authors:** Fernando Luís Scolari, Marciane Maria Rover, Geraldine Trott, Mariana Motta Dias da Silva, Denise de Souza, Raine Fogliati de Carli Schardosim, Rosa da Rosa Minho dos Santos, Emelyn de Souza Roldão, Duane Mocellin, Jennifer Menna Barreto de Souza, Aline Paula Miozzo, Gabriela Soares Rech, Carolina Rothmann Itaqui, Juliana de Mesquita, Gabriel Pozza Muller Estivalete, Hellen Jordan Martins Freitas, Catherine Vitória Pereira dos Santos, Lucas Gobetti da Luz, Marcelo Kern, Milena Soriano Marcolino, Bruna Brandão Barreto, Paulo R. Schwartzman, Ana Carolina Peçanha Antonio, Maicon Falavigna, Caroline Cabral Robinson, Carisi Anne Polanczy, Regis Goulart Rosa

**Affiliations:** 1 Porto Alegre RS Brasil Escritórios de Projetos, Hospital Moinhos de Vento - Porto Alegre (RS), Brasil.; 2 Porto Alegre RS Brasil Instituto de Pesquisa, Hospital Moinhos de Vento - Porto Alegre (RS), Brasil.; 3 Porto Alegre RS Brasil Serviço de Nefrologia, Hospital Moinhos de Vento - Porto Alegre (RS), Brasil.; 4 Porto Alegre RS Brasil Serviço de Medicina Interna, Hospital Moinhos de Vento - Porto Alegre (RS), Brasil.; 5 Belo Horizonte MG Brasil Departamento de Clínica Médica, Faculdade de Medicina, Universidade Federal de Minas Gerais - Belo Horizonte (MG), Brasil.; 6 Salvador BA Brasil Departamento de Medicina Interna e Apoio Diagnóstico, Faculdade de Medicina da Bahia, Universidade Federal da Bahia - Salvador (BA), Brasil.; 7 Porto Alegre RS Brasil Departamento de Cardiologia, Hospital Moinhos de Vento - Porto Alegre (RS), Brasil.; 8 Porto Alegre RS Brasil Unidade de Terapia Intensiva, Hospital de Clínicas de Porto Alegre, Universidade Federal do Rio Grande do Sul - Porto Alegre (RS), Brasil.; 9 Porto Alegre RS Brasil Unidade de Pesquisa, Inova Medical - Porto Alegre (RS), Brasil.

**Keywords:** COVID-19, Coronavírus, SARS-CoV-2, Síndrome do desconforto respiratório, Qualidade de vida, Unidades de terapia intensiva, Brasil

## Abstract

**Fundamento:**

As evidências acerca das sequelas a longo prazo após internação por síndrome do desconforto respiratório agudo por COVID-19 ainda são escassas.

**Objetivo:**

Avaliar alterações nas funções pulmonar, cardíaca e renal e na qualidade de vida após internação por síndrome do desconforto respiratório agudo secundária à COVID-19.

**Métodos:**

Trata-se de estudo multicêntrico, caso-controle, incluindo 220 participantes. Os casos serão definidos como pacientes hospitalizados por síndrome do desconforto respiratório agudo devido à COVID-19. No Grupo Controle, serão selecionados indivíduos sem histórico de hospitalização nos últimos 12 meses ou sintomas a longo prazo de COVID-19. Todos os indivíduos serão submetidos à espirometria pulmonar com teste de difusão pelo monóxido de carbono, tomografia de tórax, ressonância magnética cardíaca e renal com gadolínio, ergoespirometria, creatinina sérica e urinária, proteínas totais e microalbuminúria urinária, além de questionários de qualidade de vida. Os casos serão avaliados 12 meses após a alta hospitalar e os controles, 90 dias após a inclusão no estudo. Para todas as análises estatísticas, será assumido como significativo o valor p < 0,05.

**Resultados:**

O desfecho primário do estudo foi definido com a capacidade de difusão pulmonar aferida para o monóxido de carbono a partir de 12 meses. Os demais parâmetros das funções pulmonar, cardíacas e renal e da qualidade de vida foram definidos como desfechos secundários.

**Conclusão:**

Este estudo visa determinar as sequelas a longo prazo nas funções pulmonar, cardíaca e renal e na qualidade de vida de pacientes internados por síndrome do desconforto respiratório agudo devido à COVID-19 na população brasileira.

## INTRODUÇÃO

O comprometimento pulmonar causado pelo coronavírus da síndrome respiratória aguda grave 2 (SARS-CoV-2) resultou em aumento substancial na demanda por assistência ventilatória e em prolongadas admissões em unidades de terapia intensiva (UTIs).^(1)^ Dados iniciais indicam que aproximadamente 15% dos pacientes afetados pelo SARS-CoV-2 requerem hospitalização, e cerca de 3% necessitarão de cuidados intensivos.^(2)^ Em face desses números, estima-se que a doença do coronavírus 2019 (COVID-19) tenha causado mais de 12 milhões de internações em UTIs ao redor do mundo.^(3)^

A síndrome do desconforto respiratório agudo (SDRA) representa a mais temida complicação pulmonar relacionada à COVID-19 devido à sua associação com um longo tempo de internação e mortalidade.^(4)^ Aproximadamente 42% dos pacientes com pneumonia por COVID-19 desenvolveram SDRA e 60 - 80% necessitaram de cuidados intensivos.^(5)^ A SDRA é um processo inflamatório, resultante de injúrias locais ou sistêmicas na membrana alvéolo-capilar pulmonar, ocasionando incremento na permeabilidade vascular local, com subsequente edema intersticial e alveolar.^(6)^ Ainda que recebam alta hospitalar, sobreviventes de SDRA estão sob risco elevado de incapacidades físicas persistentes.^(7)^ Estudos pré-pandemia identificaram que sobreviventes de SDRA apresentam redução da função muscular, com alta prevalência de incapacidade físico-funcional, distúrbio ventilatório restritivo e diminuição da difusão pulmonar.^(8–10)^ Ainda, cerca de 39% dos sobreviventes de SDRA exibem polineuropatia do doente crítico, perpetuando a debilidade física por até 12 meses após a alta hospitalar.^(7)^ Apesar do acometimento predominantemente respiratório, diversos relatos identificam acometimento adicional no sistema cardiovascular e renal, sejam diretos ou indiretos.^(11–14)^ Esses insultos agudos podem levar a disfunções orgânicas a longo prazo, reduzindo a qualidade de vida e a necessidade de acompanhamento e tratamentos das sequelas em um período mais longo.^(15)^

De acordo com a Organização Mundial da Saúde (OMS),^(16)^ o estabelecimento de causalidade entre COVID-19 e sequelas em longo prazo configura prioridade de pesquisa. Contudo, evidências acerca da prevalência de sequelas pulmonares, cardiovasculares e renais em sobreviventes de SDRA decorrente da COVID-19 na população brasileira ainda são escassas. Apresentamos o protocolo do estudo Pós-COVID Brasil 3, com enfoque em analisar o impacto nas funções pulmonar, cardíaca e renal, além da qualidade-de-vida entre sobreviventes de SDRA por COVID-19 a partir de 12 meses após a alta.

## MÉTODOS

Trata-se de estudo caso-controle prospectivo e multicêntrico com pacientes sobreviventes à hospitalização devido à COVID-19 com diagnóstico de SDRA e controles sem sintomas persistentes de COVID-19 e hospitalização por doença clínica nos últimos 12 meses. O desenho do estudo encontra-se sumarizado na figura 1. O protocolo do estudo encontra-se registrado no Clinicaltrials.gov (NCT05225194). Este estudo foi aprovado pela Comissão Nacional de Ética em Pesquisa (Conep) (CAAE: 54943222.7.1001.5330) e adere à resolução 466/12 do Conselho Nacional de Saúde (CNS) do Brasil. Consentimento informado, livre e esclarecido será obtido de todos os pacientes.

**Figura 1:** Desenho do estudo.

SDRA - síndrome do desconforto respiratório agudo; RT-PCR - reação em cadeia da polimerase via transcriptase reversa; SARS-CoV-2 - coronavírus da síndrome respiratória aguda grave 2; PaO_2_ - pressão parcial de oxigênio; FiO_2_ - fração inspirada de oxigênio; PEEP - pressão positiva expiratória final; CPAP - pressão positiva contínua nas vias aéreas; CO_2_ - monóxido de carbono.

### Seleção de pacientes

#### Pacientes com síndrome do desconforto respiratório agudo

Pacientes serão considerados elegíveis se apresentarem idade ≥ 18 anos, teste de reação em cadeia da polimerase via transcriptase reversa (RT-PCR) positivo ou teste de antígeno para infecção por SARS-CoV-2 coletado por secreção nasofaríngea durante a internação ou até nos 14 dias anteriores, presença de SDRA devido à COVID-19 durante a hospitalização e alta hospitalar da internação índice. O diagnóstico de SDRA foi definido de acordo com os critérios de Berlim:^(17)^ insuficiência respiratória não totalmente explicada por insuficiência cardíaca ou sobrecarga hídrica; início em uma semana a partir da lesão conhecida ou de sintomas novos que se agravaram; opacidade bilaterais não totalmente explicadas por efusões, colapso lobar, pulmonar ou nódulos; hipoxemia definida por relação entre pressão parcial de oxigênio e fração inspirada de oxigênio (PaO_2_/FiO_2_) ≤ 300mmHg, com pressão positiva expiratória final ou pressão positiva contínua nas vias aéreas ≥ 5cmH_2_O. Serão excluídos indivíduos com doença de base grave com expectativa de vida inferior a 3 meses, indisponibilidade de comparecer aos exames a serem realizados, ausência de familiar responsável para casos de pacientes com dificuldade de comunicação (afasia, défice cognitivo ou não nativos em língua portuguesa) e ausência de consentimento para participação no estudo.

#### Controles

Serão considerados controles os indivíduos ≥ 18 anos, membro da família ou do convívio social de um participante sobrevivente de SDRA por COVID-19. Serão excluídos indivíduos com indisponibilidade de comparecer aos exames propostos, histórico de sintomas persistentes após infecção por SARS-CoV-2, histórico de internação não eletiva por doença clínica nos últimos 12 meses, doença de base grave com expectativa de vida inferior a 3 meses e ausência de consentimento para participação no estudo. Serão considerados sintomas persistentes da COVID-19: tosse, fadiga, dores musculares, dispneia, anosmia, ageusia, défice de memória ou concentração previamente avaliados por médico e atribuídos à COVID-19.

### Recrutamento

Os casos serão recrutados de hospitais localizados na cidade Porto Alegre (RS) e de sua respectiva região metropolitana. Os casos de SDRA serão acessados a partir de ambulatórios, de banco de dados ou de registros prévios de internação em UTI por insuficiência ventilatória de pacientes que mantêm acompanhamento clínico nos respectivos centros, e os controles serão recrutados entre os familiares ou pessoas do convívio dos casos. Serão realizados contatos telefônicos após a alta hospitalar e aplicados os critérios de elegibilidade e o Termo de Consentimento Livre e Esclarecido (TCLE). Em concordância e com consentimento devidamente esclarecido e assinado, serão realizados: a revisão de prontuário e entrevista telefônica estruturada; a teleconsulta para avaliação da elegibilidade e o agendamento dos exames previamente especificados.

### Procedimentos

Todos os participantes realizarão teleconsulta por telefone para avaliação de possíveis contraindicações para os seguintes exames complementares: creatinina e ureia séricos; creatinina e proteínas totais na urina, microalbuminúria e exame qualitativo de urina; espirometria com difusão pulmonar para o monóxido de carbono (CO_2_) sem prova broncodilatadora; ergoespirometria em esteira rolante com protocolo de rampa; tomografia de alta resolução de tórax (sem contraste) e ressonância magnética cardíaca e renal com gadolínio. Pacientes com contraindicação a um ou mais exames permanecerão no estudo, realizando os demais que forem elegíveis. A teleconsulta e os exames serão realizados a partir de 12 meses após alta hospitalar da internação índice pela SDRA, ou, ainda, no momento da inclusão dos controles (com janela restrita de até 90 dias pelo risco de os controles desenvolverem infecção por COVID-19 ou outras complicações clínicas após abordagem inicial) e com intervalo máximo de até 36 meses, a partir da alta. A figura 2 ilustra a trajetória do participante no estudo.

**Figura 2:** Trajetória do participante no estudo.

SDRA - síndrome do desconforto respiratório agudo; CO_2_ - monóxido de carbono; EQ-5D-3L - *EuroQol 5 Dimensions 3 Levels.*

### Desfechos

O desfecho primário do estudo foi definido como a capacidade de difusão pulmonar aferida para o monóxido de carbono (DLCO - *carbon monoxide diffusing capacity*) a partir de 12 meses. Foram considerados desfechos secundários: capacidade físico-funcional aferida pelo índice de Barthel modificado a partir de 12 meses;^(18)^ atividades instrumentais de vida diária aferidas pela escala de Lawton e Brody de atividades instrumentais de vida diária a partir de 12 meses;^(19)^ função e força muscular avaliadas pela *Strength, Assistance with Walking, Rising from a Chair, Climbing Stairs, and Falls* (SARC-F) a partir de 12 meses;^(20)^ dispneia aferida pela escala modificada de dispneia do *Medical Research Council* (MRC) a partir de 12 meses;^(21)^ qualidade de vida relacionada à saúde avaliada pelo questionário *EuroQol 5 Dimensions 3 Levels* (EQ-5D-3L) a partir de 12 meses;^(22,23)^ variáveis ergoespirométricas a partir de 12 meses: consumo direto de oxigênio, consumo máximo de oxigêncio (VO_2_ pico), razão volume minuto/produção de CO_2_ (VE/VCO_2_ *slope*) e eficiência de captação de oxigênio (OUES - *Oxygen Uptake Efficiency Slope*); variáveis espirométricas a partir de 12 meses: capacidade vital (CV), volume expiratório forçado no primeiro segundo (VEF1) e VEF1/CV; padrões radiológicos de fibrose pulmonar aferidos por tomografia de tórax de alta resolução a partir de 12 meses; padrões radiológicos relacionados às sequelas de miocardite aguda aferidos por ressonância magnética cardíaca com mapas paramétricos ponderados em T1 e T2 e por meio da técnica do realce tardio a partir de 12 meses; padrões radiológicos de sarcopenia aferidos por tomografia de tórax de alta resolução a partir de 12 meses; alterações de função ou estrutura renal avaliados por ressonância magnética e exames laboratoriais; e eventos cardiovasculares maiores (infarto não fatal, acidente vascular cerebral não fatal e insuficiência cardíaca aguda) e eventos tromboembólicos (trombose venosa profunda ou embolia pulmonar) após a alta hospitalar. O protocolo de coleta de dados referentes aos exames coletados encontra-se descrito no Material Suplementar.

### Seleção de centros

Os centros participantes serão selecionados após preenchimento de um questionário para avaliação de exequibilidade. Serão inclusas as instituições que demonstram alto potencial de recrutamento de participantes de pesquisa e consonância com a boas práticas clínicas, conforme as seguintes características: ser hospital referência para atendimento de casos da COVID-19 na Região Metropolitana de Porto Alegre; possuir unidade de tratamento intensivo (dez ou mais leitos) para tratamento de casos da COVID-19; demonstrar viabilidade para diagnóstico da COVID-19 por RT-PCR ou teste de antígeno; aceitar a participação no estudo por meio da assinatura de termo de cooperação.

#### Recrutamento

Os centros participantes selecionarão pacientes de acordo com seus registros internos e que preencham os critérios de inclusão para participação no estudo. O recrutamento será realizado por entrevista presencial ou remota com o paciente e/ou seu familiar pela equipe de pesquisa. Após a assinatura do TCLE e subsequente coleta de dados de linha de base, os participantes serão avaliados por meio de entrevista telefônica estruturada e centralizada. Para os casos, a entrevista será realizada a partir de 12 meses da alta hospitalar, e os controles serão avaliados imediatamente após sua inclusão no estudo com janela restrita de até 90 dias.

Os participantes serão incluídos mediante avaliação de critérios de elegibilidade, registrados em *log* de triagem. Os pacientes que apresentarem todos os critérios de inclusão e nenhum critério de exclusão serão convidados a participar voluntariamente do estudo mediante assinatura do TCLE presencial ou eletrônico. O processo de obtenção do TCLE será detalhado na seção de ética e boas práticas clínicas. A abordagem do participante será realizada por pesquisador devidamente treinado quanto às boas práticas clínicas, mantendo o anonimato e o sigilo dos dados, bem como promovendo melhores condições de conforto ao participante.

### Coleta de dados

Coleta de dados no centro participante - linha de base: aos participantes da pesquisa, será aplicada entrevista estruturada e será feita a revisão de prontuário médico para coleta de dados sociodemográficos, informações de saúde prévias, hábitos de vida anteriores à sua inclusão no estudo; para os participantes com SDRA, serão coletados dados referentes à internação por COVID-19, incluindo variáveis referentes à gravidade (*Sequential Organ Failure Assessment* [SOFA] e uso de ventilação mecânica) e tratamentos prescritos (vasopressores e corticoide).

### Acompanhamento

Todos os participantes serão acompanhados e avaliados por meio de entrevista telefônica estruturada, teleconsulta e posterior realização de exames naqueles sem contraindicações. Esse acompanhamento se realizará por contato telefônico feito por pesquisadores do centro coordenador, que é o Hospital Moinhos de Vento (HMV). Os pesquisadores receberam capacitação para a coleta de dados nessa modalidade. Os procedimentos dessa etapa serão realizados em central telefônica única, situada no HMV. Ferramentas eletrônicas (*e-mail* e *WhatsApp*) poderão ser utilizadas exclusivamente para o agendamento das entrevistas. Apenas dados de *status* vital e recusa de seguimento serão coletados por forma digital. Será realizada uma teleconsulta médica com os participantes, para avaliar possíveis contraindicações para a realização dos exames do estudo. Após realização dos exames complementares, todos participantes realizarão uma teleconsulta final para informar os resultados dos exames aos pacientes e orientações pertinentes para cada caso.

A coleta de dados será realizada com o uso de fichas clínicas eletrônicas, acessíveis por smartphone, *tablet* ou computador. O processo de coleta e gestão de dados de forma digital apresenta diversos benefícios em relação ao processo não automatizado, como a padronização, a confiabilidade e a segurança dos dados coletados. A ferramenta a ser utilizada para a coleta e gestão de dados será o *Research Electronic Data Capture* (REDCap; https://www.redcapbrasil.com.br/).^(24)^ O acesso à plataforma de dados será realizado por usuário e senha pessoais e intransferíveis, por membro da equipe, após a devida delegação no estudo pelo investigador principal. Os usuários da plataforma (equipe da pesquisa) terão permissões específicas correlatas à sua função e à delegação no estudo.

### Segurança dos dados

Diversos procedimentos serão empregados para garantir a segurança e a qualidade dos dados. Todos os pesquisadores participarão de sessão de treinamento a respeito de boas práticas clínicas e procedimentos do estudo, incluindo a coleta dos dados. Os investigadores poderão entrar em contato com o centro coordenador do estudo para resolver questões ou problemas que eventualmente surjam. Todos os processos relacionados à gestão dos dados estarão em conformidade com a Lei Geral de Proteção de Dados (lei 13.709, de 14 de agosto de 2018). Todos os servidores, *Domain Name System* (DNS) e meios de disponibilização do acesso serão construídos e mantidos em território nacional. Essa infraestrutura será dedicada ao presente estudo. Portanto, todos os dados serão isolados de qualquer outro acesso, garantindo maior segurança. Além disso, o fato de os servidores estarem sediados em território nacional submeterá a custódia desses dados à legislação brasileira. Serão utilizados, ainda, certificados digitais para criptografia, para garantir a segurança das comunicações.

O processo de* backup* do banco de dados será feito de forma automatizada com periodicidade de 12 horas. A extração de dados para o *software* estatístico se dará de forma automatizada, com anonimização dos dados para processos de checagem de consistência de dados, ações de monitoria remota, desenvolvimento de variáveis derivadas e análises estatísticas. Limpezas de dados para identificação de inconsistências serão conduzidas periodicamente. Os pesquisadores serão notificados sobre as inconsistências, para que providenciem correção. As entrevistas telefônicas serão gravadas e auditadas para verificação de consistência dos dados. Os arquivos de áudio serão armazenados de forma anonimizada em servidor, com o mesmo sistema de segurança do banco de dados descrito. O acesso a esses arquivos ocorrerá de forma protegida, por usuário e senha pessoais e intransferíveis, pela equipe de pesquisa. O centro coordenador revisará mensalmente relatórios detalhados sobre triagem, inclusão, seguimento, consistências e completude dos dados. Tomará imediatamente ações para resolver eventuais problemas. Técnicas estatísticas para identificação de fraude serão realizadas ao longo do estudo.

### Cálculo de tamanho amostral

Foi calculado um tamanho de amostra de 198 indivíduos (99 para cada grupo) para testar se existe uma diferença mínima clinicamente relevante de 2mL/minute/mmHg nas médias de DLCO entre os grupos SDRA COVID e controle saudáveis. Considerou-se acréscimo de 10% para possíveis perdas e recusas, somando 110 para cada grupo, considerando poder de 80%, nível de significância de 5% e desvio-padrão igual a 5mL/minuto/mmHg.

### Análise estatística

A descrição de variáveis será realizada no formato de frequência absoluta e relativa para variáveis qualitativas, no formato de média e desvio-padrão para variáveis quantitativas simétricas e no formato de mediana e intervalo interquartil para variáveis quantitativas assimétricas. A distribuição de variáveis quantitativas será averiguada por visualização gráfica de histogramas e teste de Shapiro-Wilk. O desfecho primário será comparado entre os grupos de estudo utilizando regressão logística ajustada por sexo, idade e comorbidades. A medida de efeito reportada será a razão de chances (RC) com intervalo de confiança de 95% (IC95%). Desfechos secundários com distribuição qualitativa serão avaliados da mesma forma como o desfecho primário. Desfechos secundários com distribuição quantitativa serão avaliados por meio de regressão linear múltipla ajustada por sexo, idade e comorbidades. A medida de efeito reportada será a diferença de médias com intervalo de confiança de 95%. Valor de alfa < 0,05 será considerado estatisticamente significativo. Desfechos secundários não serão ajustados para multiplicidade. Dessa forma, os achados em desfechos secundários serão interpretados como exploratórios. As análises serão realizadas com o *software* R, na versão a ser detalhada no momento da análise.^(25)^

### Ética e boas práticas clínicas

A pesquisa foi planejada e será realizada em conformidade com a resolução 466 de 12 de dezembro de 2012 do CNS^(26)^ e das Diretrizes de Boas Práticas Clínicas, Emenda 6 - Revisão 2 do *International Council for Harmonisation*,^(27)^ além das normas preconizadas pela Lei Geral de Proteção de Dados. A pesquisa será iniciada apenas após aprovação completa do protocolo e documentos correlatos pelo sistema Comitê de Ética em Pesquisa/Conep nos locais de condução do estudo.

### Consentimento para participação

Seguindo as Diretrizes e Normas Regulamentadoras de Pesquisa Envolvendo Seres Humanos, estabelecidos pela resolução 466/12 do CNS, um TCLE será apresentado aos participantes da pesquisa no momento do convite para entrar no estudo. O TCLE será elaborado de maneira que forneça, em linguagem acessível e clara, as informações acerca dos objetivos, da metodologia, do processo de coleta e do registro das informações do estudo. O TCLE poderá ser aplicado de forma presencial (em pacientes que estejam em consulta de revisão) ou eletrônica.

### Disseminação

Os pesquisadores submeterão os resultados do estudo em congressos e reuniões científicas e escreverão manuscrito que será submetido para publicação em periódico revisado por pares. O comitê diretivo do estudo participará da análise dos resultados, da elaboração do manuscrito e da seleção do periódico para publicação. Os critérios de autoria serão definidos de acordo com o *International Committee of Medical Journal Editors* (ICMJE).

### Compartilhamento de dados

Os autores encorajam pesquisadores a contatarem o autor correspondente para acesso e compartilhamento de dados não publicados. O comitê diretivo terá papel em julgar e fornecer os dados requisitados.

## DISCUSSÃO

As sequelas a longo prazo entre pacientes sobreviventes de SDRA devido à COVID-19 ainda são pouco conhecidos, e sua magnitude poderá impactar no planejamento e no aporte de recursos pelos sistemas de saúde. Dados provenientes de coorte italiana de paciente internados em UTI por SDRA no primeiro semestre de 2020 demonstraram que, dentre os sobreviventes, um terço apresentou sinais de fadiga e perda de força muscular 3 meses após a alta, com melhora do componente físico na qualidade de vida após 12 meses.^(7)^ A avaliação pulmonar realizada 3 meses após a alta, em outra coorte, revelou alta prevalência de alterações funcionais e estruturais pulmonares.^(28)^ Contudo, esses dados refletem, em sua maioria, dados de coortes europeias ou norte-americanas com infecção grave por COVID-19, sobretudo de pacientes inclusos nas primeiras ondas da doença, antes da vacinação da população. Ainda é desconhecida a prevalência de sequelas na saúde física e mental entre brasileiros sobreviventes de SDRA por COVID-19.

Eventos cardiovasculares têm sido relatados durante a fase aguda da COVID-19,^(13)^ mas o risco pode persistir após a resolução da infecção. Estudo populacional sugere que esses indivíduos estão sob risco de apresentar eventos cardiovasculares, como acidente vascular cerebral, arritmias, síndrome coronariana aguda, miocardite e pericardite até 1 ano após a fase aguda.^(14)^ Estudos com ressonância magnética cardíaca demonstraram alta prevalência de alterações miocárdicas pós-COVID. Contudo, a maioria desses estudos inclui pacientes atendidos ambulatorialmente e com seguimento de apenas 4 meses.^(29)^ Nosso estudo pretende determinar a prevalência de sequelas cardíacas a longo prazo entre indivíduos com COVID-19 grave como dilatação ventricular e com perda de função cardíaca. Ainda, a detecção de fibrose miocárdica por meio da técnica do realce tardio por gadolínio pode estar relacionada à miocardite prévia ou a eventos isquêmicos na fase aguda da COVID-19. Sua detecção está relacionada a eventos arrítmicos e pior prognóstico,^(30)^ sendo importante na estratificação da gravidade desses indivíduos.

A injúria renal aguda é uma complicação comum na fase aguda da COVID-19 e está associada a piores desfechos e evolução para doença renal crônica, principalmente naqueles com maior gravidade na apresentação.^(12)^ Os fatores que podem estar associados a injúria renal pela COVID-19 envolvem dano endotelial mediados por agente viral, ativação de complemento, inflamação local e glomerulopatia,^(11)^ assim como outros fatores, como estado hemodinâmico na infecção aguda, medicação nefrotóxica e eventos trombóticos, entre outros agentes indiretos.^(31)^ O dano renal a longo prazo pode estar relacionado à manutenção direta do agente agressor, mas também de forma indireta, pela exacerbação de doenças cardiovasculares que levam a maior dano renal.^(11)^ Esperamos poder estimar a prevalência de dano renal associado à COVID-19 em longo prazo na população brasileira por meio de mensuração de função renal e de análise estrutural.

Este estudo possui diversos pontos fortes. Trata-se de estudo multicêntrico com avaliação prospectiva de sequelas a longo prazo. Muitos dos estudos prévios que investigaram COVID-19 longa e suas sequelas incluíram pacientes em um período curto após a infecção aguda.^(13,28)^ Contudo, devido à redução do número de casos e da gravidade da COVID-19, os pacientes inclusos poderão corresponder a fases mais iniciais da pandemia. Além disso, a coleta retrospectiva de dados referentes à internação índice pode limitar nosso estudo.
